# A Computational Study of Heteroatom Analogues of Selenoxide and Selenone *syn* Eliminations

**DOI:** 10.3390/molecules29204915

**Published:** 2024-10-17

**Authors:** Adrian I. Doig, Jessica T. Stadel, Thomas G. Back

**Affiliations:** Department of Chemistry, University of Calgary, Calgary, AB T2N 1N4, Canada

**Keywords:** organoselenium chemistry, DFT computations, selenoxides, selenones, *syn* eliminations, heteroatom *syn* eliminations, zwitterions

## Abstract

Selenoxide *syn* elimination is a widely used method for the synthesis of alkenes because it proceeds under exceptionally mild conditions, typically with excellent regio- and stereoselectivity. Surprisingly, hetero-selenoxide eliminations, where one or both olefinic carbon atoms are replaced with heteroatoms, have been little investigated, and their selenonyl counterparts even less so. A variety of such reactions, where the heteroatoms included combinations of O, N and S, as well as C, were investigated computationally. Selenoxides typically have lower activation energies and are slightly endothermic, while the corresponding selenones display higher activation energies and are exothermic in the gas state. The results are consistent with concerted, five-centre processes, leading to the formation of dioxygen, aldehydes, diazenes and imines from seleninyl or selenonyl peroxides, esters, hydrazines and amines, respectively. The more acidic selenenyl hydrodisulfide analogue undergoes proton transfer to the basic selenoxide oxygen atom instead of concerted elimination, resulting in the formation of a zwitterion. However, the formation of the corresponding selenonyl zwitterion is disfavoured compared to concerted *syn* elimination. The effects of solvents were also computed along with changes in enthalpy, entropy and free energy. Solvent effects were variable, while free energy calculations indicated overall ΔG values ranging between 3.60 and −32.12 kcal mol^−1^ for the syn eliminations of methyl methanethioseleninate and methaneperoxyselenonic acid, respectively. These computations suggest that the olefin-forming selenoxide *syn* elimination may be more general than currently understood and that replacement of the two carbon atoms with heteroatoms can lead to viable processes.

## 1. Introduction

The 1,2 eliminations that afford alkenes are among the most fundamental and widely used processes in synthetic organic chemistry. *Syn* eliminations are a subset that typically proceed by concerted, unimolecular processes [1] involving five- or six-centred transition states and afford complementary stereochemistry to that of *E*_2_ eliminations. Early examples of *syn* processes include ester pyrolyses, as well as Chugaev eliminations of xanthates and Cope eliminations of amine oxides. Unfortunately, these reactions typically require high temperatures that make them unsuitable for many applications. The related sulfoxide elimination has also been employed in alkene synthesis, but it is similarly hampered by the requirement for elevated temperatures. In contrast, selenoxide *syn* eliminations (for selected reviews, see [2,3,4,5]; for seminal papers, see [6,7,8,9,10]) often proceed readily at room temperature and are typically highly regio- and stereoselective. Moreover, the introduction of the required selenium residue into an organic substrate can be easily achieved via either electrophilic, nucleophilic or radical processes, thus further enhancing the popularity of this useful reaction.

The widely employed alkyl selenoxide elimination has been studied previously by computational methods and is included here for comparison with the heteroatom analogues that are the focus of the present investigation. For example, early work by Kwart et al. [11] demonstrated that tunnelling plays an important role in lowering the activation energies of these processes compared to those of other *syn* eliminations. A computational investigation of the regioselectivity of selenoxide eliminations by Fujimoto et al. [12] indicated that oxygen and nitrogen substituents resulted in the preferential formation of allylic ether, alcohol and amine products, while delocalizing groups such as cyano substituents afforded vinylic products exclusively. They also reported that the transition states were asynchronous, with earlier hydrogen transfer compared with retarded C-Se bond cleavage. Schiesser and coworkers [13] performed computations that revealed that substituents on α-arylseleno ketones had little effect on elimination rates and that the oxidation of selenium was generally rate-determining in the overall process. Selenoxide eliminations of selenocysteine derivatives were modelled by Bayse and Allison [14], who reported that increased chain lengths in RSeCys (vs. R = Me or Ph) decreased activation energies, as did *ortho* Lewis base substituents (when R = aryl) that stabilized the transition state by electron donation to the positive selenium centre. More recently, Orian and coworkers [15] reported a rigorous computational study, including a comparison with analogous sulfoxides, telluroxides and eliminations via their higher oxidation states. These researchers found that the activation energies decreased in the order S > Se > Te in chalcogenoxide eliminations and were higher in the selenonyl analogues compared to their selenoxides. The facile hydration of telluroxides impeded their ability to eliminate. Furthermore, activation energies were correlated with the basicities of the chalcogenoxide oxygen atoms and the chalcogen–carbon bond strengths. Their research included studies of biologically relevant selenocysteine derivatives.

**Scheme 1:**
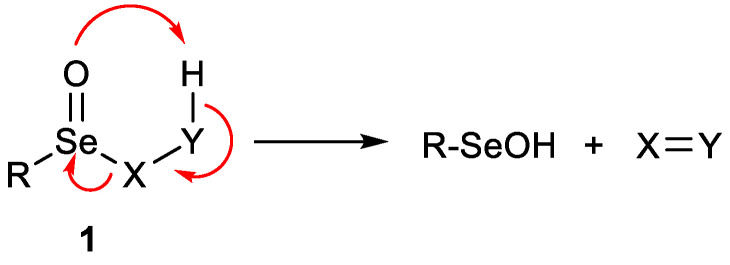
Hetero-selenoxide *syn* eliminations.

**Scheme 2:**
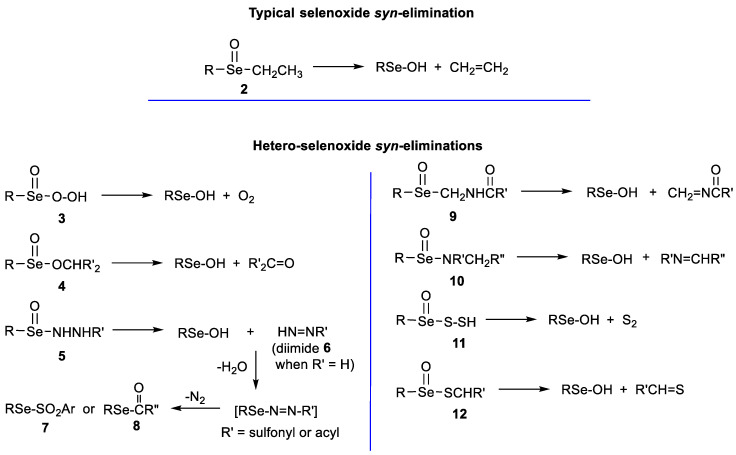
Examples of known and potential hetero-selenoxide *syn* eliminations.

**Scheme 3:**
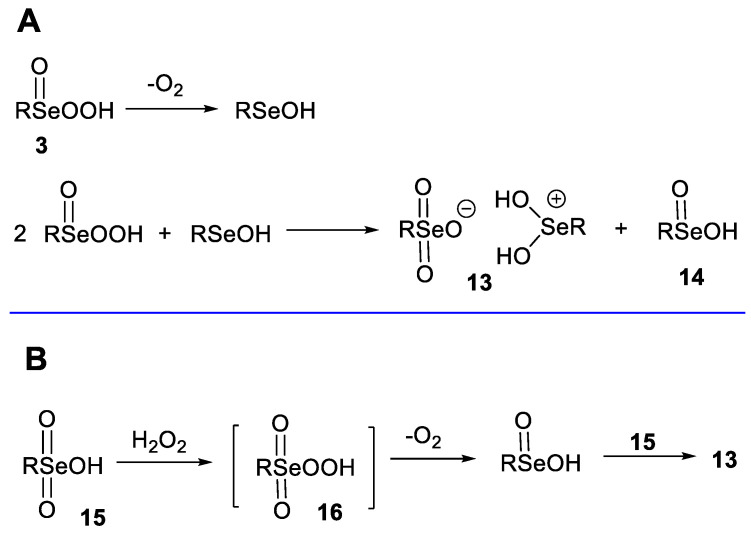
(**A**) Formation of dioxygen by elimination of seleninic peroxy acids. (**B**) Formation of dioxygen during the reaction of selenonic acids with hydrogen peroxide.

In contrast with the well-known and synthetically useful selenoxide *syn* elimination for the preparation of alkenes, to our knowledge, there has been no systematic computational study of related processes where either or both of the two alkene-generating carbon atoms are replaced by heteroatoms (X and Y in **1** in Scheme 1). Several such reactions, in addition to the alkene-forming process of selenoxide **2**, are illustrated in Scheme 2 for selenoxide analogues **3**–**12**, which are of interest for a variety of reasons. For example, during recent studies of selenium-catalyzed epoxidations of alkenes, it was discovered that peroxyseleninic acids **3** decompose to the corresponding selenonium selenonate salts **13**, with the concomitant evolution of dioxygen [16] (Scheme 3, path A). The vigorous liberation of oxygen was also reported during the preparation of selenonic acids **15** in the presence of hydrogen peroxide [17] (Scheme 3, path B). The salt **13** (R = Ph), containing mixed oxidation states of selenium, had been previously misidentified as the selenonic acid **14** [18], but its structure was unequivocally established by X-ray crystallography [16]. It was therefore of interest to ascertain whether or not selenoxide eliminations of peroxy acids **3** or their selenonyl analogues **16** could be responsible for the observed formation of oxygen, by analogy with the formation of alkenes from alkyl selenoxides **2**. The oxidations of alcohols to ketones with benzeneseleninic anhydride and its congeners have also been reported, most likely via the elimination of seleninate ester intermediates **4** [19,20,21]. 

Similarly, the oxidation of hydrazines with seleninic acids or anhydrides presumably involves the formation of *N*-(seleninyl)hydrazines **5**, which afford diimide **6** when R’ = H [22] (Scheme 2). Alternatively, in substituted hydrazines, further reaction of the intermediate diazenes with the byproduct selenenic acid, followed by the loss of dinitrogen, affords selenosulfonates **7** [23] or selenoesters **8** [24]. The conversion of amines and amides to the corresponding imines via selenoxides **9** [25] or by the treatment of amines with benzeneseleninic anhydride and related oxidants via postulated seleninamides **10** has also been reported [26,27,28], as was the similar oxidation of primary amines to nitriles [26]. 

Furthermore, the generation of diatomic sulfur (S_2_) from silyl or germanyl trisulfides was reported by Steliou, Gareau and Harpp et al. [29], who trapped it by its Diels–Alder cycloaddition with dienes. Although the multiplicity of S_2_ was not established, the authors noted that the ground state of this species is a triplet, while the energy of the corresponding singlet state is 13 kcal mol^−1^ higher. The possibility that diatomic sulfur could be produced from seleninyl hydrodisulfides **11** by selenoxide elimination remains to be explored. Finally, the similar oxidation and elimination of thioseleninates **12** (Scheme 2) was first reported by Reich and Jasperse [30], and later by Glass et al. [31] and by us [32] as a step in the mechanism by which the antioxidant drug ebselen catalyzes the reduction of peroxides with sacrificial thiols, accompanied by the formation of the corresponding thioaldehyde. In each case in Scheme 2, the unstable byproduct selenenic acid (RSeOH) undergoes disproportionation, reduction, oxidation or reaction with nucleophiles, depending on the specific conditions. Since the mechanisms reported for most of the reactions shown in Scheme 2 are speculative, a computational study of these postulated hetero-selenoxide eliminations appeared warranted. The corresponding selenonyl analogues of selenoxides **1** and their possible role in such processes have been much less studied and so were included in this investigation. Thus, in order to gain insight into the viability of such reactions and their kinetic and thermodynamic properties, we now report geometry optimization and transition-state computations of a variety of hetero-*syn* eliminations of species of general structure **1** and their Se(VI) analogues in both the gaseous state and in the presence of solvents.

## 2. Results

In principle, if X and Y in selenoxide structure **1** are confined to C, O, N and S, then sixteen structures are possible, along with sixteen others based on the corresponding selenonyl analogues that can undergo known or hypothetical *syn* elimination processes. Table 1 includes the computed values of activation energies (ΔE^‡^) and overall energy changes (ΔE) for sixteen of these processes that correspond to synthetically useful or theoretically interesting transformations, as summarized in Scheme 2. For consistency, ease of comparison and simplicity of computation, all examples are based on methyseleno derivatives. 

With respect to computations in the gaseous state, entries 1 and 2 in Table 1 correspond to an alkyl selenoxide and selenone elimination, respectively, and are included for comparison. These computations indicate that the selenoxide reacts slightly endothermically, while the selenone reacts exothermically, but with a higher activation energy. The analogous eliminations of the peroxyseleninic and peroxyselenonic acids are shown in entries 3 and 4. The mechanism for dioxygen generation in the above processes was unclear, but the possibility of a selenoxide *syn* elimination was adumbrated [16]. The salts **13** were the products of protonation of the amphoteric seleninic acids **14** by the stronger selenonic acids **15**, which were in turn formed by the redox equilibration of selenium species of different oxidation state (Scheme 3). Furthermore, while the peroxyseleninic acid in entry 3 of Table 1 reacts with a relatively high activation energy in an endothermic process, the corresponding peroxyselenonic acid in entry 4 has a similar activation energy (29.1 kcal mol^−1^) to that of alkene formation in entry 2 (30.7 kcal mol^−1^) and is exothermic overall. The lower activation energy for peroxyselenonic acid (29.1 vs 36.4 kcal mol^−1^) and its more exothermic reaction energy (−13.7 vs. 4.9 kcal mol^−1^) compared to its seleninic counterpart indicate that the process in entry 4 is more facile than that in entry 3. These results cast doubt on the facile liberation of dioxygen from the peroxyseleninic acids **3** at or below room temperature, as shown in Scheme 3A, via a concerted *syn* elimination and suggest that further oxidation to the selenonic species **15** and **16** may be required for this process. The more facile *syn* elimination of oxygen from postulated peroxyacids **16**, produced from the oxidation of selenonic acids **15** with hydrogen peroxide, would again generate salts **13**, as shown in Scheme 3B [33]. 

The oxidations of alcohols with benzeneseleninic anhydride [PhSe(=O)]_2_O likely produce intermediate seleninate esters **4** (Scheme 2) that in turn fragment to afford aldehydes or ketones. The results shown in entry 5 of Table 1 reveal a slightly exothermic process with an activation energy of 26.6 kcal mol^−1^ for the transformation of the methyl seleninate to formaldehyde, indicating that a *syn* elimination is a viable mechanism for this type of oxidation. Interestingly, the reaction of the corresponding selenonate ester (entry 6) is more exothermic, but it is kinetically disfavoured with a higher activation energy of 42.5 kcal mol^−1^.

Hydrazines are readily oxidized by seleninic acids or anhydrides via the formation of postulated seleninyl hydrazine intermediates **5**, as shown in Scheme 2, followed by their spontaneous *syn* elimination. Thus, the unsubstituted hydrazine (R’ = H) generated diimide **6**, which was confirmed by the in situ transfer hydrogenation of cinnamic acid to hydrocinnamic acid and azobenzene to hydrazobenzene [22]. When substituted hydrazines were employed, the resulting azenes reacted further with the selenenic acid byproduct, followed by the extrusion of dinitrogen to afford selenosulfonates **7** or selenoesters **8**, as shown in Scheme 2. The selenoxide elimination of intermediate **5** is characterized by a lower activation energy and moderate reaction energy (10.1 and 5.1 kcal mol^−1^, respectively; Table 1, entry 7) for the formation of *trans*-diimide, while higher values of 11.4 and 7.0 kcal mol^−1^ were obtained for the formation of *cis*-diimide. The elimination of the selenonyl analogue of **5** was again characterized by a higher activation energy and a significantly more exothermic reaction (entry 8).

*Syn* eliminations are also plausible mechanisms for the conversion of α-aminoalkyl selenoxides to imines, as shown in Table 1, entry 9 (X = CH_2_, Y = NH), and in the transposed seleninamide in entry 11 (X = NH, Y = CH_2_). Like the hydrazine in entry 7, these systems also comprise slightly endothermic processes. However, the seleninamide reaction in entry 11 possesses a considerably higher activation energy than that in entry 9 (23.8 vs. 8.2 kcal mol^−1^), attributed in part to the greater acidity of the amino moiety compared to that of the methyl group in the proton transfer step to the basic selenoxide oxygen atom. Again, the corresponding selenone systems in entries 10 and 12 display higher activation energies and more exothermic reactions than their selenoxide counterparts.

**Figure 1:**
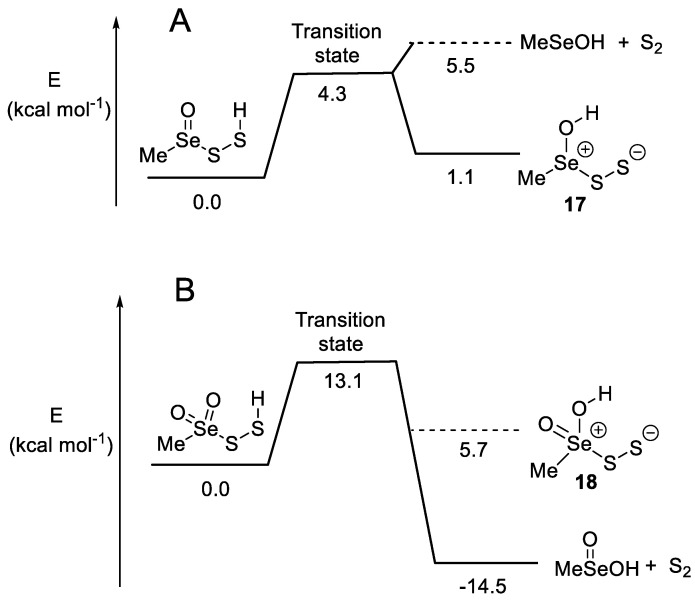
Activation and reaction energies (kcal mol^−1^} for seleninyl (**A**) and selenonyl (**B**) hydrodisulfides in the gaseous state.

**Scheme 4:**
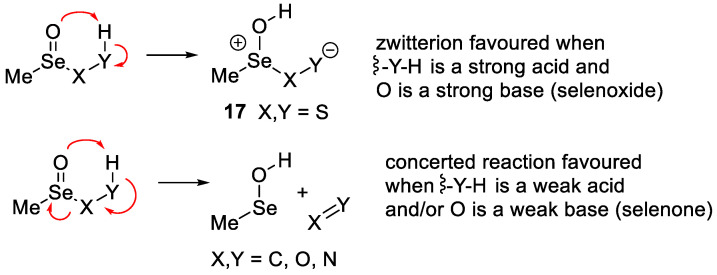
Zwitterion formation and concerted elimination reactions of hetero-selenoxides.

The reactions of seleninyl and selenonyl hydrodisulfides are summarized in Table 1, entries 13 and 14, respectively. The seleninyl compound revealed anomalous behaviour, where computations indicated an activation energy of 4.3 kcal mol^−1^, which is actually lower than that of the sum of the presumed fragmentation products (MeSeOH and S_2_) [34,35] (Scheme 4). However, IRC calculations revealed that proton transfer from the hydrodisulfide moiety to the selenoxide oxygen atom was more advanced than cleavage of the Se-S bond, suggesting the possible formation of zwitterion **17** instead of elimination products, as shown in Scheme 4. Indeed, geometry optimization of **17** indicated that it has an energy 3.2 kcal mol^−1^ lower than that of the transition state and is only 1.1 kcal mol^−1^ higher than the starting hydrodisulfide (Figure 1A). This can be attributed to the considerably higher acidity of the S-S-H moiety (pKa 6–7 [36]) compared to the β-hydrogens (C-H, O-H or N-H) of the other starting materials in Table 1, which facilitates proton transfer. Further evidence for the formation of **17** stems from a comparison of key interatomic distances in the transition state and products (Table 2; vide infra). Thus, it appears that in the case of the seleninyl hydrodisulfide, the proton transfer leads to a discrete zwitterion intermediate instead of to a merely asynchronous concerted elimination. This was confirmed by the corresponding IRC computation. 

In contrast, the selenonyl hydrodisulfide provided a calculated exothermic reaction energy of −14.5 kcal mol^−1^ and a higher activation energy of 13.1 kcal mol^−1^, in contrast to the seleninyl derivative (Table 1, entry 14). Moreover, the energy of the corresponding zwitterion product **18** was 20.2 kcal mol^−1^ higher in energy than that of its *syn* elimination products (Figure 1B). Since the selenonyl hydrodisulfide is expected to be even more acidic than the corresponding selenoxide hydrodisulfide, we attribute the difference in behaviour, at least in part, to the lower basicity of the selenone oxygen atom [37,38] and the weaker Se-S bond when the selenium atom is in a higher oxidation state. In entry 14, the S_2_ product was assumed to be in the triplet ground state [35], but even if the less stable singlet state was formed, the products would still be lower in energy than the zwitterion. It therefore appears that in this case, *syn* elimination would be the preferred pathway. 

Finally, the thioseleninate and thioselenonate in Table 1, entries 15 and 16, displayed activation energies of 18.3 and 27.0 kcal mol^−1^, respectively, which are slightly lower than those in entries 1 and 2. The former compound also provided the most endothermic elimination in Table 1, with a reaction energy of 17.1 kcal mol^−1^. This can be attributed to the instability of both the thioformaldehyde and selenenic acid products. 

The results shown in Table 1 also include solvent effects from water, dichloromethane and methanol. In the case of the conventional selenoxide elimination (*n* = 1, X,Y = CH_2_) and its selenone counterpart (entries 1 and 2), both the activation and overall energies increased in the presence of the solvents, indicating that solvation stabilized the starting materials more strongly than the corresponding transition states and products. The same was noted for entries 7 and 9. The solvents lowered the activation energies in entries 3, 4, 8, 10, 13 and 14, relative to their gaseous states, and raised them in the remaining entries 5, 6, 11, 12, 15 and 16. The overall changes in energy were rendered more exothermic or were insignificant in the presence of the solvents in the latter entries. 

**Figure 2:**
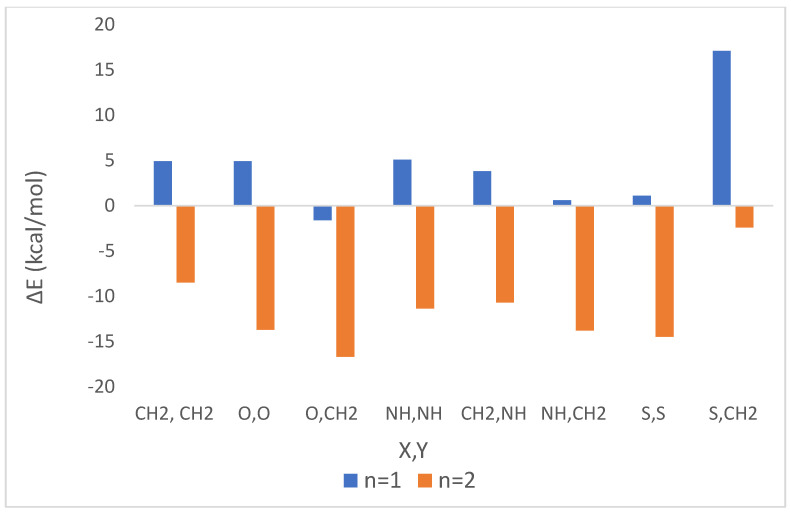
Reaction energies ΔE for heteroatom selenoxide and selenone *syn* eliminations in the gaseous state. All results represent concerted *syn* eliminations, except for the seleninyl hydrodisulfide (*n* = 1, X,Y = S), where the product is zwitterion **17**.

**Figure 3:**
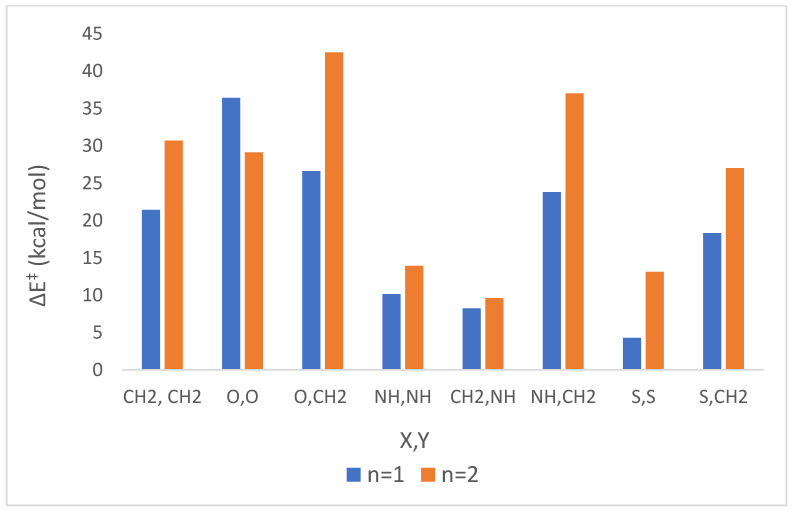
Activation energies ΔE^‡^ for heteroatom selenoxide and selenone *syn* eliminations in the gaseous state. All results represent concerted *syn* eliminations, except for the seleninyl hydrodisulfide (*n* = 1, X,Y = S), where the product is zwitterion **17**.

The overall results of the gas-state computations are summarized in Figure 2 and Figure 3, illustrating the reaction energies (ΔE) and activation energies (ΔE^‡^) for the processes in Table 1. It is striking that all of the selenonyl eliminations are exothermic, while their selenoxide counterparts are endothermic, except in the case of the methyl seleninate (*n* = 1; X = O, Y = CH_2_). On the other hand, activation energies are higher for selenones compared to selenoxides, except for the peroxides (X,Y = O). Thus, in general, the concerted eliminations of selenoxides are kinetically favoured over the corresponding selenone eliminations, while the opposite is true for their overall thermodynamic outcomes.

Key bond lengths and interatomic distances in starting materials and transition states are provided in Table 2. As expected, the transition states typically show elongation of the X-Se and proton-accepting Se=O bonds, contraction of the X-Y bond and the partial transfer of H from Y to O. However, in entries 3, 13 and 14 of Table 2, the X-Se bond is actually slightly shorter in the transition state than in the starting material, while the S-S bond in entries 13 and 14 is slightly longer. Furthermore, the H---O=Se separation shows a dramatic decrease from 3.142 Å to 1.328 Å for the selenoxide in entry 13 and from 3.262 Å to 1.243 Å for the selenone in entry 14. Hydrogen migration in the transition state is indicated by the relative H---O=Se and H---Y distances and is most advanced (closer to O=Se than to Y) in entries 1, 3, 4 and 13–16. An asynchronous concerted reaction, as noted earlier for the alkene-forming selenoxide elimination in entry 1 [12], is also postulated for the selenonyl hydrodisulfide; however, the formation of a discrete zwitterion intermediate is ruled out on the basis of its thermodynamic instability.

The thermochemical values of ΔG, ΔH and ΔS for the conversion of starting materials to transition states and final products are provided in Table S1 on p. 17 of the Supporting Information. All reactions were exothermic, except for the formation of zwitterion **17** in entry 13 and the elimination of the thioseleninate in entry 15, which were slightly endothermic. Electron densities in transition states were calculated with Mulliken electron charges by summing and comparing the charges for the XY and seleninic or selenonic acid components. The results are shown in Table S2 on p. 17 of the Supporting Information, which revealed that electron density [39] resides preferentially on the XY component of the transition states, except in the *syn* elimination of ethyl methyl selenoxide in entry 1, where both components were essentially neutral. 

## 3. Methods

Computations were performed using the DFT B3LYP platform in Gaussian 2016 [40]. A mixed basis set was employed, using 6-311G(d,p) for C, H, N, O and S, and the cc-pVTZ triple zeta variation of Dunning’s correlation-consistent basis set for Se [41]. Transition-state energies were computed by means of the Berny algorithm. All transition states except for the hydroperoxide in Table 1, entry 3, resulted in single imaginary frequencies involving hydrogen migration and partial cleavage of the X-Se bond, while geometry optimizations of products and starting materials showed no imaginary frequencies. IRC computations were performed on all transition states (forward and backward pathways), resulting in agreement with the original geometry optimizations, except in the case of the hydroperoxide in entry 3 [42]. Enthalpies of the reactions were calculated as the difference in the sums of the electronic and thermal enthalpies of the products and reactants. Gibbs free energies of the reactions were calculated similarly, using the sums of electronic and thermal free energies. Computations were performed on the Advanced Research Computing (ARC) computer cluster at the University of Calgary.

## 4. Conclusions and Summary

Figure 2 and Figure 3 clearly show that in all cases except for the peroxy species (entries 3 and 4 in Table 1), the selenoxide eliminations in the gas state produced significantly lower activation energies than their selenonyl analogues. Furthermore, all of the selenoxide reactions, except for the seleninate ester in entry 5 in Table 1, were endothermic, while all of the selenone eliminations were exothermic. These observations are parallel to those made by Orian et al. [15] in the *syn* eliminations of oxidized selenocysteine and related compounds. The asynchronous nature of the alkyl selenoxide eliminations that was noted previously by Fujimoto et al. [12] applies similarly to the heteroatom-substituted systems in the present work, where proton transfer from Y to O in the transition states advances more rapidly than X-Se cleavage. In the extreme case of the seleninyl hydrodisulfide in entry 13 of Table 1, facile proton transfer produces a discrete intermediate in the form of zwitterion **17**, instead of the concerted fragmentation to MeSeOH and S_2_. Solvent effects varied considerably in the examples in Table 1, increasing activation energies in some instances and decreasing them in others, attributed to the preferential solvation of starting materials and transition states, respectively. Enthalpies, entropies and free energies were also obtained, and ΔG values indicated exothermic reactions for all examples except entries 13 and 15, which were slightly endothermic. Most of the selenoxide eliminations in Table 1 have been little studied, except for entry 1, while their selenonyl counterparts remain virtually unexplored. In a more general sense, these results demonstrate that several extensions of the classical olefin-forming selenoxide elimination to systems containing heteroatoms instead of simple alkyl substituents are kinetically and thermodynamically viable, except where acidic hydrogens are transferred to strongly polarized Se=O bonds, resulting in the initial formation of discrete zwitterion intermediates.

## Figures and Tables

**Table 1:** Electronic energies in hetero-selenoxide *syn* eliminations ^a,b^.

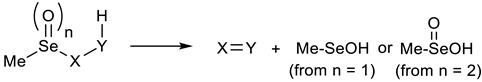											
Entry	n	X	Y	ΔE^‡^ Gas Phase	ΔEGas Phase	ΔE^‡^ H_2_O	ΔE H_2_O	ΔE^‡^ CH_2_Cl_2_	ΔE CH_2_Cl_2_	ΔE^‡^ MeOH	ΔE MeOH
1	1	CH_2_	CH_2_	21.4	4.9	24.3	7.4	23.8	7.0	24.2	7.3
2	2			30.7	−8.5	31.4	−6.0	31.3	−6.4	31.4	−6.1
3 ^c,d^	1	O	O	36.4	4.9	32.5	7.5	33.2	7.1	32.6	7.4
4 ^c^	2			29.1	−13.7	28.7	−12.9	28.8	−12.9	28.7	−12.9
5	1	O	CH_2_	26.6	−1.6	28.1	−2.5	27.9	−2.3	28.1	−2.4
6	2			42.5	−16.7	42.7	−19.2	42.7	−18.7	42.7	−19.1
7 ^e^	1	NH	NH	10.1	5.1	12.3	6.0	11.9	5.8	12.2	6.0
8 ^e^	2			13.9	−11.4	9.9	−11.9	11.5	−11.8	10.1	−11.8
9	1	CH_2_	NH	8.2	3.8	10.1	5.0	9.8	4.8	10.1	5.0
10	2			9.6	−10.7	5.0	−9.2	6.2	−9.4	5.3	−9.2
11	1	NH	CH_2_	23.8	0.6	26.4	0.6	26.0	0.6	26.3	0.6
12	2			37.0	−13.8	37.8	−15.3	37.7	−15.0	37.8	−15.2
13 ^f^	1	S	S	4.3	1.1	3.7	−1.6	3.8	−1.1	3.7	−1.5
14	2			13.1	−14.5	12.3	−13.9	12.5	−14.0	12.4	−13.9
15	1	S	CH_2_	18.3	17.1	19.7	17.0	19.4	17.0	19.6	17.0
16	2			27.0	−2.4	28.3	−3.2	28.1	−3.1	28.3	−3.2

Legend for Table 1. ^a^ ΔE^‡^ = Activation energy. ^b^ ΔE = Overall change in energy between the starting material and products in the reaction. ^c^ Based on the formation of triplet O_2_. ^d^ These energies may be overestimated, as rotation of the methyl group was frozen to avoid additional imaginary frequencies. ^e^ Based on the formation of *trans*-diimide (**6**). ^f^ Based on the formation of zwitterion **17**.

**Table 2:** Key interatomic distances (Å) in starting materials and transition states in the gaseous state ^a^.

		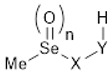					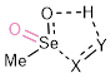				
		Starting Material					Transition State				
Entry	*n*, X, Y	X-Se	X-Y	H-OSe	H-Y	Se=O	X-Se	X-Y	H-OSe	H-Y	Se=O
1	1, C, C	2.005	1.521	2.492	1.093	1.651	2.481	1.415	1.278	1.334	1.707
2	2, C, C	1.966	1.524	2.995 ^b^	1.091	1.618, 1.618	2.513	1.415	1.384	1.278	1.689, 1.629
3	1, O, O	1.858	1.443	2.332	0.975	1.630	1.824	1.426	1.180	1.298	1.999
4	2, O, O	1.831	1.446	2.744	0.971	1.605, 1.600	2.055	1.337	1.103	1.376	1.707, 1.615
5	1, O, CH2	1.818	1.435	2.420	1.093	1.629	2.283	1.308	1.310	1.300	1.685
6	2, O, CH2	1.775	1.446	2.950 ^b^	1.092	1.607, 1.607	2.237	1.317	1.370	1.278	1.621, 1.682
7	1, NH, NH	1.940	1.418	2.190	1.027	1.647	2.499	1.314	1.334	1.180	1.708
8	2, NH, NH	1.883	1.414	2.425 ^c^	1.014	1.613, 1.617	2.581	1.292	1.700	1.059	1.677, 1.633
9	1, CH2, NH	2.074	1.421	2.214	1.023	1.660	2.530	1.335	1.341	1.181	1.715
10	2, CH2, NH	2.029	1.420	3.006	1.014	1.622, 1.622	2.641	1.320	1.632	1.072	1.682, 1.634
11	1, NH, CH2	1.885	1.475	2.554	1.096	1.642	2.370	1.370	1.310	1.303	1.693
12	2, NH, CH2	1.840	1.474	2.870 ^d^	1.093	1.613, 1.612	2.370	1.371	1.372	1.281	1.684, 1.628
13	1, S, S	2.371	2.086	3.142	1.352	1.636	2.338	2.094	1.328	1.555	1.684
14	2, S, S	2.324	2.086	3.262	1.352	1.612, 1.612	2.290	2.105	1.243	1.634	1.677, 1.607
15	1, S, CH2	2.330	1.830	2.190	1.096	1.642	2.631	1.699	1.182	1.417	1.710
16	2, S, CH2	2.279	1.837	2.356	1.090	1.617, 1.610	2.576	1.705	1.247	1.375	1.697, 1.620

Legend for Table 2. ^a^ When the hydrogen source contains several hydrogen atoms, the H-Y distance is listed for the closest hydrogen to the Se=O oxygen. ^b^ The two respective Y-H---O=Se distances were equal. ^c^ The H-bonded (Y)NH---O=Se distance is indicated; the shortest (X)NH---O=Se distance was 2.683 Å. ^d^ The (X)NH---O=Se distance was 2.613 Å.

## Data Availability

The original contributions presented in the study are included in the article and Supplementary Materials. Further inquiries can be directed to the corresponding author.

## Supplementary Materials

The following supporting information can be downloaded at https://www.mdpi.com/article/10.3390/molecules29204915/s1: Energies, Z-coordinates, numbers of imaginary frequencies and depictions of optimized structures of starting materials (pp. 2–8), transition states (pp. 9–15) and zwitterion products (p. 15), along with overall free energies and free energies of activation (Thermochemistry, Table S1, p. 17); electron densities in transition states (Table S2, p. 18).
