# Supplementary material for: A Computational Study of Heteroatom Analogues of Selenoxide and Selenone syn Eliminations
Source: Molecules. 2024 Oct 17;29(20):4915. doi: 10.3390/molecules29204915 (PMC11510669; doi:10.3390/molecules29204915)
Supplement: Supplementary file 1 [file molecules-29-04915-s001.zip › molecules-3159167-supplementary.pdf]

SUPPORTING INFORMATION

for

A Computational Study of Heteroatom Analogues of Selenoxide and Selenone *syn*-Eliminations

Adrian I. Doig, Jessica T. Stadel and Thomas G. Back*

Contents:

Computations of Starting Materials p. 2-8

Computations of Transition States p. 9-15

Computations of Zwitterions p. 15

Table S1. Thermochemistry p. 17

Table S2. Electron Density in Transition States p. 18

Computations of Starting Materials

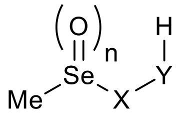

Ethyl methyl selenoxide (n = 1; X, Y = CH_2)

E(RB3LYP) = -2596.00201446 a.u.; imaginary frequencies = 0

Symbolic Z-matrix:
Charge = 0 Multiplicity = 1

C	-0.73135	-1.65971	0.32788
H	-1.78393	-1.82564	0.55285
H	-0.13957	-1.73833	1.23767
H	-0.3904	-2.35602	-0.43985
Se	-0.58617	0.21295	-0.31273
O	-0.65983	1.10474	1.09586
C	1.37911	0.16175	-0.72009
C	2.21146	-0.1755	0.50691
H	1.57476	1.17458	-1.07751
H	1.5164	-0.5337	-1.55235
H	3.26269	0.07267	0.33591
H	1.85633	0.40539	1.36223
H	2.15669	-1.23636	0.75886

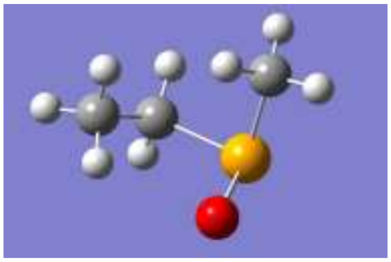

Ethyl methyl selenone (n = 2; X, Y = CH_2)

E(RB3LYP) = -2671.20360481 a.u.; imaginary frequencies = 0

Symbolic Z-matrix:
Charge = 0 Multiplicity = 1

C	1.78883	1.14341	0.00793
H	2.7017	0.54983	0.01127
H	1.71421	1.74567	0.91189
H	1.72187	1.74622	-0.89633
Se	0.32462	-0.14399	-0.00006
O	0.36075	-0.94962	1.40247
C	-1.28986	0.97804	-0.01366
C	-2.52921	0.09104	0.00115
H	-1.21449	1.58756	-0.91608
H	-1.21164	1.61209	0.87153
H	-3.42829	0.71212	-0.00198
H	-2.55361	-0.55565	-0.87754
H	-2.5456	-0.53626	0.89394
O	0.38429	-0.95549	-1.39837

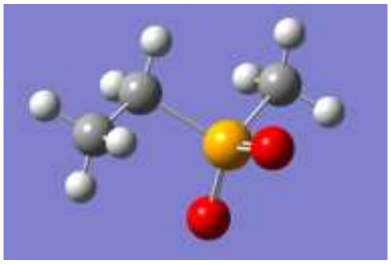

Methaneperoxyseleninic acid (n = 1; X, Y = O)

```
E(RB3LYP) =  -2667.75288729 a.u.; imaginary frequencies = 0

Symbolic Z-matrix:
 Charge =  0 Multiplicity = 1
 C                    -0.74375  -1.58456   0.41748
 H                    -1.70431  -1.60428   0.93193
 H                     0.08057  -1.73079   1.11266
 H                    -0.71459  -2.30632  -0.39878
 Se                   -0.52138   0.21999  -0.31552
 O                    -0.45256   1.14999   1.02104
 O                     1.27147   0.01702  -0.76075
 O                     2.01178  -0.27603   0.44238
 H                     1.88215   0.54126   0.95563
```

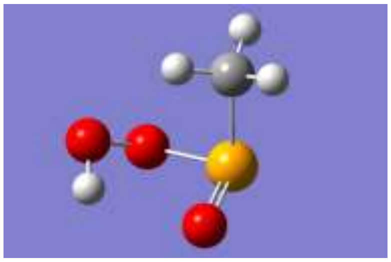

Methaneperoxyselenonic acid (n = 2; X, Y = O)

```
E(RB3LYP) =  -2742.94608520 a.u.; imaginary frequencies = 0

Symbolic Z-matrix:
 Charge =  0 Multiplicity = 1
 C                     0.28491   1.84359  -0.01653
 H                     1.06179   2.21638   0.64831
 H                    -0.71115   2.10683   0.33119
 H                     0.45586   2.1344   -1.05051
 Se                    0.3885   -0.09198   0.05086
 O                     0.19779  -0.59223   1.56432
 O                    -1.15387  -0.52049  -0.83851
 O                    -2.24861   0.05622  -0.09084
 H                    -2.42507  -0.64018   0.56182
 O                     1.5422   -0.66246  -0.90007
```

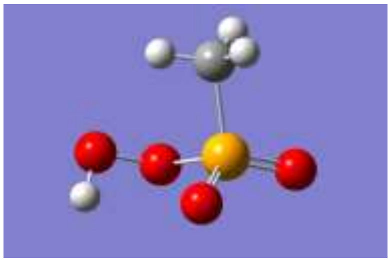

Methyl methaneseleninate (n = 1; X = O, Y = CH_2)

```
E(RB3LYP) =  -2631.91409002 a.u.; imaginary frequencies = 0

Symbolic Z-matrix:
 Charge =  0 Multiplicity = 1
 C                    -0.95961  -1.51797   0.4585
 H                    -2.03298  -1.51068   0.65172
 H                    -0.41127  -1.60429   1.39574
 H                    -0.69476  -2.31024  -0.24161
 Se                   -0.49959   0.23027  -0.33231
 O                    -0.49836   1.21053   0.96906
 C                     2.13877  -0.18636   0.41798
 H                     1.80759   0.50101   1.20124
 H                     2.22446  -1.19934   0.82486
 H                     3.11047   0.13603   0.04153
 O                     1.23682  -0.16249  -0.69829
```

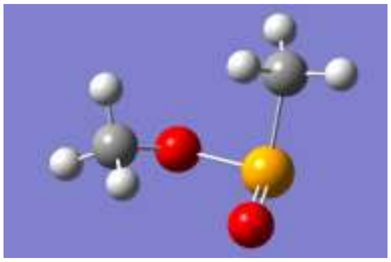

Methyl methaneselenonate (n = 2; X = O, Y =CH_2)

E(RB3LYP) = -2707.11293928 a.u.; imaginary frequencies = 0

Symbolic Z-matrix:
Charge = 0 Multiplicity = 1

C	-1.5999	1.27926	-0.00008
H	-2.58314	0.81263	0.00055
H	-1.43127	1.8609	-0.90338
H	-1.43045	1.86163	0.90259
Se	-0.3123	-0.16237	0.00001
O	-0.35287	-0.9698	-1.38855
C	2.38594	0.22657	-0.00002
H	3.13225	1.01954	0.00016
H	2.49632	-0.38736	0.89634
H	2.49651	-0.38721	-0.89647
O	1.10843	0.90257	-0.00006
O	-0.35286	-0.96958	1.38869

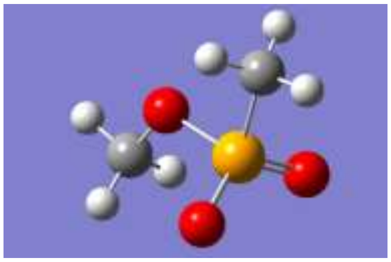

(*N*)-Methylseleninylhydrazine (n = 1; X, Y = NH)

E(RB3LYP) = -2628.05485641 a.u.; imaginary frequencies = 0

Symbolic Z-matrix:
Charge = 0 Multiplicity = 1

C	1.39078	1.30531	-0.25882
H	0.67044	2.11745	-0.17151
H	1.8929	1.33316	-1.22631
H	2.11036	1.33735	0.55866
Se	0.41345	-0.39162	-0.08931
O	-0.09508	-0.38065	1.47959
H	-1.94248	0.30581	0.94816
N	-1.25677	0.30663	-0.9458
H	-1.68241	-0.54765	-1.30291
N	-2.12306	0.78948	0.05719
H	-1.90909	1.7712	0.20392

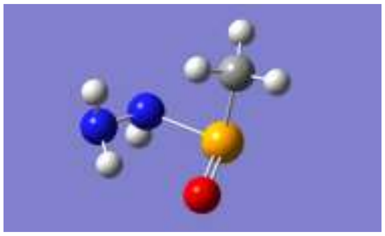

(*N*)-Methylselenonylhydrazine (n = 2; X, Y = NH)

E(RB3LYP) = -2703.25790955 a.u.; imaginary frequencies = 0

Symbolic Z-matrix:
Charge = 0 Multiplicity = 1

C	0.87247	0.62507	1.91711
H	0.03284	1.1216	2.3976
H	1.72343	1.28494	1.76526
H	1.15617	-0.28392	2.44588
Se	0.27444	-0.06351	0.16757

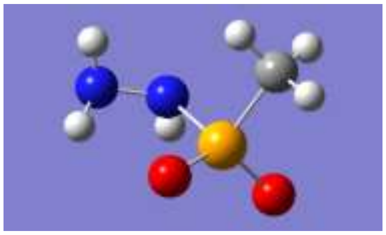

O	-1.00886	-0.99648	0.56014
H	-2.12789	-0.43739	-1.24219
H	-2.64145	1.10167	-0.82268
N	-0.77632	1.05723	-1.42619
H	-0.23408	0.53617	-2.11919
N	-2.03342	0.56388	-1.4279
O	1.57651	-0.7785	-0.49268

Aminomethyl methyl selenoxide (n = 1; X = CH_2, Y = NH)

E(RB3LYP) = -2612.04454164 a.u.; imaginary frequencies = 0

Symbolic Z-matrix:
Charge = 0 Multiplicity = 1

C	1.07758	1.43825	0.55165
H	2.01789	1.24926	1.0674
H	0.30326	1.69097	1.27638
H	1.20046	2.22697	-0.19271
Se	0.55349	-0.24628	-0.33745
O	0.23645	-1.27117	0.92937
C	-1.40363	0.33209	-0.70901
H	-1.72156	-0.37885	-1.47277
H	-1.40202	1.3432	-1.11598
N	-2.17598	0.20558	0.47708
H	-1.88551	-0.63946	0.97568
H	-2.03472	0.98952	1.10513

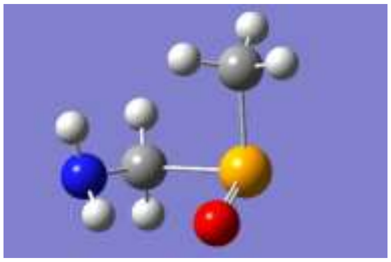

Aminomethyl methyl selenone (n = 2; X = CH_2, Y = NH)

E(RB3LYP) = -2687.24430310 a.u.; imaginary frequencies = 0

Symbolic Z-matrix:
Charge = 0 Multiplicity = 1

C	1.85458	1.0832	-0.00143
H	2.7351	0.44237	-0.00967
H	1.81924	1.68385	0.90605
H	1.8101	1.69337	-0.90223
Se	0.31925	-0.13055	0.00016
O	0.33321	-0.95022	1.39988
C	-1.37423	0.98812	-0.00153
H	-1.31288	1.60065	-0.90126
H	-1.31214	1.60343	0.89637
N	-2.5246	0.15604	-0.00045
H	-2.57051	-0.43042	0.82617
H	-2.56998	-0.4331	-0.82517
O	0.33388	-0.955	-1.39675

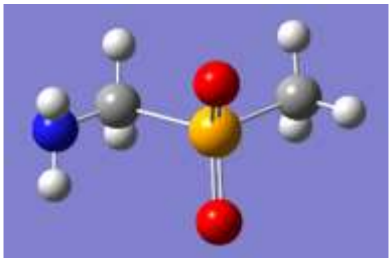

(*N*)-Methyl methaneseleninamide (n = 1; X = NH, Y = CH_2)

```
E(RB3LYP) =  -2612.03938475 a.u.; imaginary frequencies = 0

Symbolic Z-matrix:
 Charge =  0 Multiplicity = 1
 C                    -0.87726  -1.57593   0.39132
 H                    -1.94785  -1.63589   0.58526
 H                    -0.32535  -1.67148   1.32533
 H                    -0.567    -2.31825  -0.34289
 Se                   -0.52782   0.22579  -0.32081
 O                    -0.57951   1.16976   1.0212
 C                     2.1607   -0.17298   0.47111
 H                     1.90424   0.58846   1.21614
 H                     2.1038   -1.15705   0.94087
 H                     3.19049  -0.02116   0.14133
 N                     1.28023  -0.13638  -0.71167
 H                     1.56122   0.62853  -1.32097
```

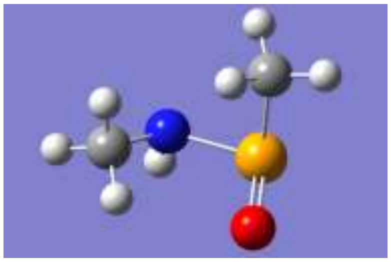

(*N*)-Methyl methaneselenonamide (n = 2; X = NH, Y = CH_2)

```
E(RB3LYP) =  -2687.23934944 a.u.; imaginary frequencies = 0

Symbolic Z-matrix:
 Charge =  0 Multiplicity = 1
 C                    -1.33104   1.48388   0.33276
 H                    -2.2979    1.38767  -0.15832
 H                    -0.76416   2.32293  -0.06518
 H                    -1.43572   1.5413    1.41403
 Se                   -0.34271  -0.13443  -0.07043
 O                    -0.02481  -0.15357  -1.65136
 C                     2.45754   0.20322   0.02827
 H                     2.32043   0.8836   -0.81087
 H                     3.26172   0.58803   0.65936
 H                     2.74578  -0.77395  -0.3702
 N                     1.22654   0.17606   0.84003
 H                     1.25557  -0.54147   1.56082
 O                    -1.07249  -1.37051   0.66619
```

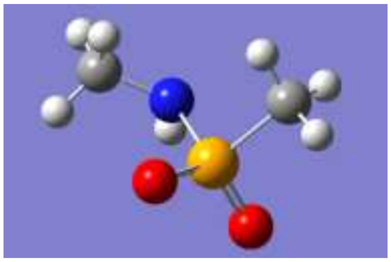

Methaneseleninyl hydrodisulfide (n = 1; X, Y = S)

```
E(RB3LYP) =  -3313.80168313 a.u.; imaginary frequencies = 0

Symbolic Z-matrix:
 Charge =  0 Multiplicity = 1
 C                    -1.06515   1.36726   1.04832
 H                    -2.00396   1.88123   0.84143
 H                    -0.22142   1.95695   0.69144
```

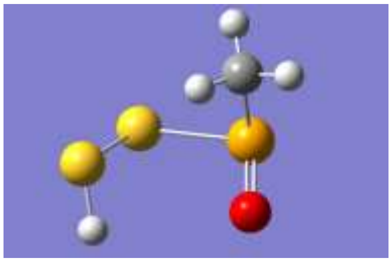

H	-0.97089	1.12135	2.10563
Se	-1.10537	-0.29041	-0.01121
O	-1.04649	0.27466	-1.54507
S	1.10166	-0.97673	0.50407
S	2.23292	0.61546	-0.23172
H	2.1885	0.29385	-1.54416

Methaneselenonyl hydrodisulfide (n = 2; X, Y = S)

E(RB3LYP) = -3388.99267761 a.u.; imaginary frequencies = 0

Symbolic Z-matrix:
Charge = 0 Multiplicity = 1

C	1.16882	-1.13818	1.42533
H	2.0965	-1.6733	1.22634
H	0.32473	-1.8202	1.50385
H	1.25671	-0.47975	2.28648
Se	0.87048	-0.00853	-0.13807
O	0.66305	-0.97253	-1.4137
S	-1.13926	1.06751	0.31754
S	-2.53423	-0.44848	-0.00651
H	-2.60115	-0.36576	-1.35428
O	1.9732	1.16675	-0.14835

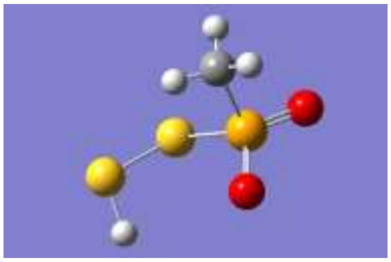

Methyl methanethioseleninate (n = 1; X = S, Y = CH_2)

E(RB3LYP) = -2954.90951050 a.u.; imaginary frequencies = 0

Symbolic Z-matrix:
Charge = 0 Multiplicity = 1

C	-1.36733	-1.05305	1.09616
H	-2.42098	-0.81644	1.25023
H	-0.78088	-0.78593	1.97354
H	-1.23304	-2.10142	0.83103
Se	-0.74106	0.07359	-0.39979
O	-0.67061	1.56582	0.28328
C	2.14501	0.55807	0.752
H	1.3914	1.34421	0.88279
H	2.37645	0.09277	1.70893
H	3.04096	0.99	0.30894
S	1.47006	-0.67386	-0.41985

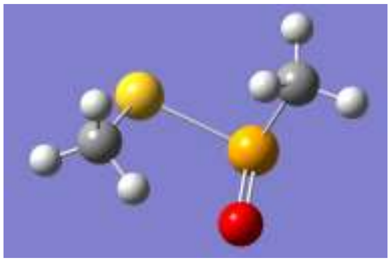

Methyl methanethioselenonate (n = 2; X = S, Y = CH_2)

```
E(RB3LYP) =  -3030.10135165 a.u.; imaginary frequencies = 0

Symbolic Z-matrix:
 Charge =  0 Multiplicity = 1
 C                      1.36808   0.20949   1.62064
 H                      2.37396  -0.205     1.56655
 H                      0.75277  -0.31697   2.34683
 H                      1.38621   1.28297   1.79646
 Se                     0.58038  -0.0787   -0.13928
 O                      0.36417  -1.67091  -0.32777
 C                     -2.51991  -0.6157    0.14125
 H                     -1.9125   -1.48016  -0.1308
 H                     -2.90432  -0.72433   1.15382
 H                     -3.33703  -0.50706  -0.57029
 S                     -1.48026   0.89379   0.01406
 O                      1.4487    0.7663   -1.20012
```

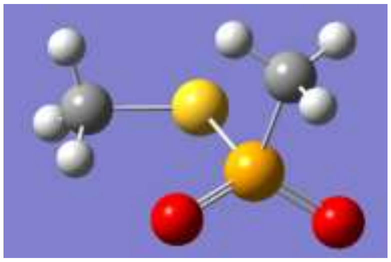

Computations of Transition States

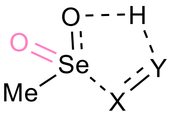

Ethyl methyl selenoxide (n = 1; X, Y = CH_2)

E(RB3LYP) = -2595.96794505 a.u.; imaginary frequencies = 1

Symbolic Z-matrix:
Charge = 0 Multiplicity = 1

C	1.9176	0.53796	1.59713
H	1.99776	0.19627	2.60794
H	1.46243	1.50621	1.58298
H	2.89334	0.59615	1.16187
Se	0.82846	-0.70355	0.57929
O	-1.23965	-1.38542	0.26627
H	-2.13199	-0.34277	-0.53203
C	0.43889	0.87492	-1.18612
C	-0.89909	0.52133	-1.40685
H	0.99605	0.19907	-1.55397
H	1.00319	1.51422	-0.76737
H	-1.10471	1.41989	-1.17703
H	-0.91952	0.08764	-2.25183

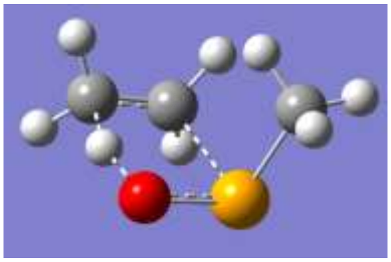

Ethyl methyl selenone (n = 2; X, Y = CH_2)

E(RB3LYP) = -2671.15464953 a.u.; imaginary frequencies = 1

Symbolic Z-matrix:
Charge = 0 Multiplicity = 1

C	1.82923	0.21	2.11663
H	1.70254	-0.19597	3.09849
H	1.60614	1.25637	2.13181
H	2.84064	0.06552	1.79871
Se	0.62963	-0.68917	0.88535
O	-0.9729	-0.39577	1.14521
H	-1.18206	0.22093	-0.18429
C	0.54985	0.4419	-1.74867
C	-0.82176	0.77017	-1.69719
H	0.79553	-0.25457	-2.52292
H	1.19049	1.29014	-1.8711
H	-0.99045	1.82195	-1.79816
H	-1.38657	0.26937	-2.45554
O	1.02434	-2.25017	0.91472

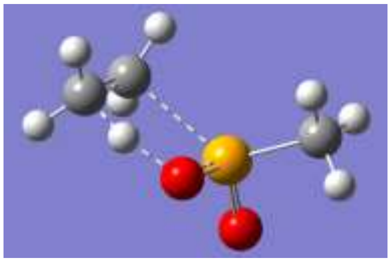

Methaneperoxyseleninic acid (n = 1; X, Y = O)

```
E(RB3LYP) =  -2667.69493911 a.u.; imaginary frequencies = 1
(H-C-Se-O dihedral angle was frozen at 180° to prevent rotation of CH3
group)

Symbolic Z-matrix:
 Charge =  0 Multiplicity = 1
 C(Fragment=1)    -1    1.36518  -0.05448  -0.28538
 H(Fragment=1)    0     1.93136  -0.80531   0.2251
 H(Fragment=1)    0     1.00762   0.66479   0.42152
 H(Fragment=1)    -1    1.98763   0.43298  -1.00639
 Se(Fragment=2)   -1   -0.14162  -0.88104  -1.18535
 O(Fragment=2)    -1   -0.95705  -1.61675   0.48612
 O(Fragment=2)    0    -1.33561   0.61368  -0.76317
 O(Fragment=2)    0    -2.62054  -0.17119  -0.47538
 H(Fragment=2)    0    -2.08205  -0.38717   0.625
```

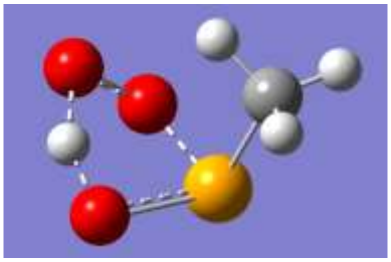

Methaneperoxyselenonic acid (n = 2; X, Y = O)

```
E(RB3LYP) =  -2742.89969698 a.u.; imaginary frequencies = 1

Symbolic Z-matrix:
 Charge =  0 Multiplicity = 1
 C                     1.32306   1.01336   0.82339
 H                     1.89604   0.55934   1.60472
 H                     0.70337   1.78189   1.23599
 H                     1.98405   1.43932   0.09775
 Se                    0.20936  -0.32919  -0.02562
 O                     1.20552  -1.44634  -0.61978
 O                    -0.98129  -0.93034   0.91633
 O                    -1.56952   0.63736  -1.82561
 O                    -2.56493   0.24536  -1.22083
 H                    -1.99844  -0.42604  -0.02468
```

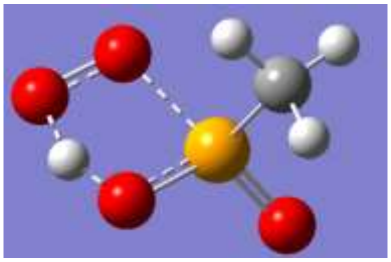

Methyl methaneseleninate (n = 1; X = O, Y = CH_2)

```
E(RB3LYP) =  -2631.87174788 a.u.; imaginary frequencies = 1

Symbolic Z-matrix:
 Charge =  0 Multiplicity = 1
 C                     1.78663   0.3615    1.84918
 H                     1.8649   -0.26928   2.70992
 H                     1.22018   1.23471   2.09723
 H                     2.7657    0.65063   1.52867
 Se                    0.89663  -0.60224   0.41995
 O                    -0.68882  -0.97339   0.68705
 H                    -1.13667  -0.22821  -0.47055
 C                    -0.99578   0.69771  -1.79023
```

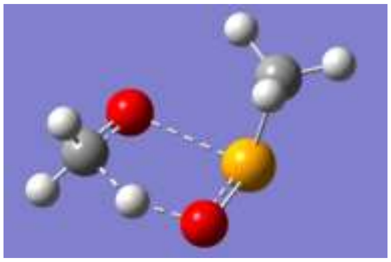

H	-1.52051	1.62487	-1.69058
H	-1.35401	0.20425	-2.66948
O	0.30803	0.86574	-1.81233

Methyl methaneselenonate (n = 2; X = O, Y = CH_2)

E(RB3LYP) = -2707.04525598 a.u.; imaginary frequencies = 1

Symbolic Z-matrix:
Charge = 0 Multiplicity = 1

C	1.72374	0.21162	2.15764
H	1.64617	-0.34077	3.07075
H	1.34601	1.2016	2.30649
H	2.74958	0.26263	1.8577
Se	0.68933	-0.67428	0.77606
O	-0.9315	-0.66752	1.04375
H	-1.1657	0.08223	-0.18256
C	-0.78342	0.85868	-1.56257
H	-1.07841	1.88694	-1.53901
H	-1.23366	0.40035	-2.41821
O	1.30374	-2.15238	0.59971
O	0.52949	0.72061	-1.56106

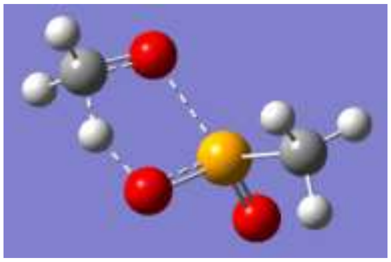

(*N*)-Methylseleninylhydrazine (n = 1; X, Y = NH)

E(RB3LYP) = -2628.06118166 a.u.; imaginary frequencies = 1

Symbolic Z-matrix:
Charge = 0 Multiplicity = 1

C	1.39078	1.30531	-0.25882
H	0.67044	2.11745	-0.17151
H	1.8929	1.33316	-1.22631
H	2.11036	1.33735	0.55866
Se	0.41345	-0.39162	-0.08931
O	-0.09508	-0.38065	1.47959
H	-1.94248	0.30581	0.94816
N	-1.25677	0.30663	-0.9458
H	-1.68241	-0.54765	-1.30291
N	-2.12306	0.78948	0.05719
H	-1.90909	1.7712	0.20392

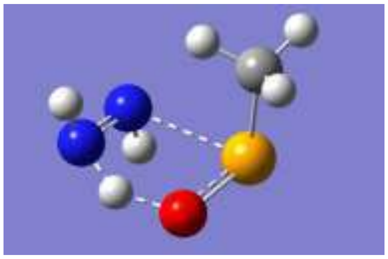

(*N*)-Methylselenonylhydrazine (n = 2; X, Y = NH)

E(RB3LYP) = -2703.23572455 a.u.; imaginary frequencies = 1

Symbolic Z-matrix:
Charge = 0 Multiplicity = 1

C	1.78663	0.3615	1.84918
H	1.8649	-0.26928	2.70992
H	1.22018	1.23471	2.09723
H	2.7657	0.65063	1.52867
Se	0.89663	-0.60224	0.41995
O	-0.68882	-0.97339	0.68705
H	-1.13667	-0.22821	-0.47055
H	-1.52051	1.62487	-1.69058
N	0.30803	0.86574	-1.81233
H	0.80908	0.55081	-2.61841
N	-0.99578	0.69771	-1.79023
O	1.68028	-1.61388	-0.43246

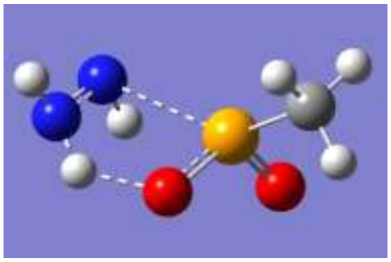

Aminomethyl methyl selenoxide (n = 1; X = CH_2, Y = NH)

E(RB3LYP) = -2612.03140777 a.u.; imaginary frequencies = 1

Symbolic Z-matrix:
Charge = 0 Multiplicity = 1

C	0.98424	-1.90566	1.08397
H	0.56818	-2.51098	1.86203
H	1.86594	-1.41811	1.44427
H	1.23498	-2.52334	0.24701
Se	-0.31458	-0.57223	0.53752
O	-0.68533	0.61521	1.63998
H	-0.25303	1.74578	0.7914
C	0.58537	1.45071	-1.29065
H	0.03203	1.48642	-2.20576
H	1.62026	1.37169	-1.55074
N	0.3113	2.45624	-0.45047
H	0.44236	3.43689	-0.59587

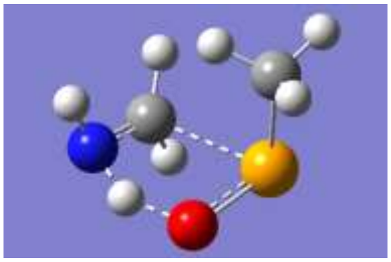

Aminomethyl methyl selenoxide (n = 2; X = CH_2, Y = NH)

E(RB3LYP) = -2687.22898778 a.u.; imaginary frequencies = 1

Symbolic Z-matrix:
Charge = 0 Multiplicity = 1

C	-0.58187	-1.55159	1.34692
H	-1.61257	-1.8203	1.44866
H	-0.25045	-1.06443	2.24008
H	0.00214	-2.43319	1.18379
Se	-0.37287	-0.34923	-0.16114

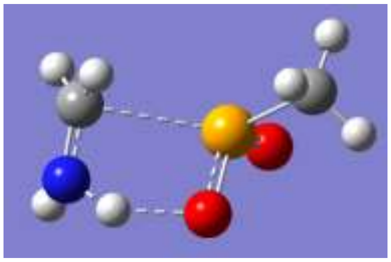

```
 O                      -1.05559   1.1449     0.03256
 H                       0.24168   1.86666   -0.09606
 C                       2.23207   0.79329   -0.44621
 H                       2.69234   0.6063    -1.39389
 H                       2.93009   0.48726    0.3048
 O                      -0.88857  -1.15242   -1.45819
 N                       1.77022   2.04738   -0.29438
 H                       2.29197   2.9004    -0.30671
```

(N)-Methyl methaneseleninamide (n = 1; X = NH, Y = CH_2)

```
E(RB3LYP) =  -2612.00150805 a.u.; imaginary frequencies = 1

Symbolic Z-matrix:
 Charge =  0 Multiplicity = 1
 C                       1.87847   0.36093    1.81214
 H                       1.90149  -0.14149    2.75657
 H                       1.45934   1.33738    1.93773
 H                       2.87398   0.44664    1.42937
 Se                      0.7917   -0.65391    0.5661
 O                      -0.83394  -0.73143    0.89621
 H                      -1.25134  -0.1059    -0.36364
 C                      -1.06319   0.58796   -1.82966
 H                      -1.44761   1.58579   -1.86778
 H                      -1.52636   0.02801   -2.61504
 N                       0.27246   0.54642   -1.8839
 H                       0.90036   0.8567    -2.59768
```

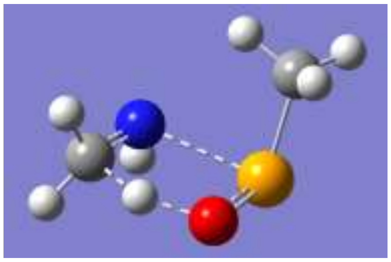

(N)-Methyl methaneselenonamide (n = 2; X = NH, Y = CH_2)

```
E(RB3LYP) =  -2687.18032200 a.u.; imaginary frequencies = 1

Symbolic Z-matrix:
 Charge =  0 Multiplicity = 1
 C                       1.74245   0.19992    2.1842
 H                       1.66046  -0.35123    3.09766
 H                       1.36683   1.19105    2.33071
 H                       2.76938   0.2479     1.88754
 Se                      0.7102   -0.68481    0.80025
 O                      -0.92477  -0.66429    1.03955
 H                      -1.18207   0.09171   -0.20336
 C                      -0.81666   0.87629   -1.60251
 H                       1.22375   1.02558   -2.21273
 H                      -1.11265   1.90442   -1.58711
 H                      -1.26375   0.4178    -2.45971
 O                       1.32889  -2.16236    0.63446
 N                       0.51709   0.72331   -1.57298
```

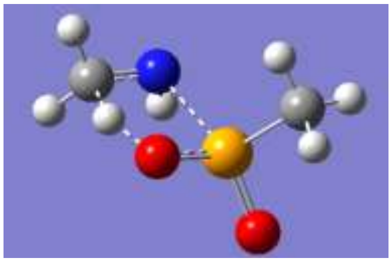

Methaneseleninyl hydrodisulfide (n = 1; X, Y = S)

E(RB3LYP) = -3313.79480204 a.u.; imaginary frequencies = 1

Symbolic Z-matrix:
Charge = 0 Multiplicity = 1

C	2.3639	1.65814	1.28195
H	2.55611	1.92223	2.30087
H	1.92622	2.49192	0.77386
H	3.28284	1.3911	0.80326
Se	1.14283	0.15176	1.22365
O	-0.3936	0.46979	1.79975
H	-1.17906	0.17042	0.60513
S	-1.59019	-0.37076	-1.25828
S	0.14373	-0.68333	-1.75476

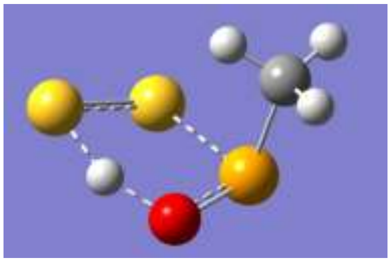

Methaneselenonyl hydrodisulfide (n = 2; X, Y = S)

E(RB3LYP) = -3388.97171325 a.u.; imaginary frequencies = 1

Symbolic Z-matrix:
Charge = 0 Multiplicity = 1

C	1.05333	1.02943	1.34661
H	2.07012	0.70016	1.39794
H	0.62707	1.02516	2.32803
H	1.01944	2.02157	0.94735
Se	0.04412	-0.16467	0.19804
O	-1.52137	0.33885	-0.07112
H	-1.45218	0.49242	-1.52725
S	0.99446	-0.08471	-2.90293
O	0.12772	-1.63807	0.84262
S	-0.68848	0.48138	-3.36501

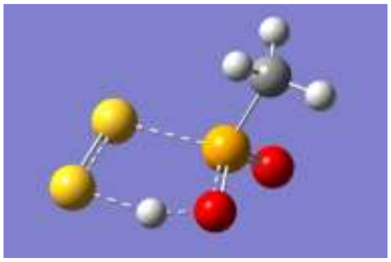

Methyl methanethioseleninate (n = 1; X = S, Y = CH_2)

E(RB3LYP) = -2954.88040689 a.u.; imaginary frequencies = 1

Symbolic Z-matrix:
Charge = 0 Multiplicity = 1

C	1.78663	0.3615	1.84918
H	1.8649	-0.26928	2.70992
H	1.22018	1.23471	2.09723
H	2.7657	0.65063	1.52867
Se	0.89663	-0.60224	0.41995
O	-0.68882	-0.97339	0.68705
H	-1.13667	-0.22821	-0.47055
C	-0.99578	0.69771	-1.79023
H	-1.52051	1.62487	-1.69058
H	-1.35401	0.20425	-2.66948
S	0.30803	0.86574	-1.81233

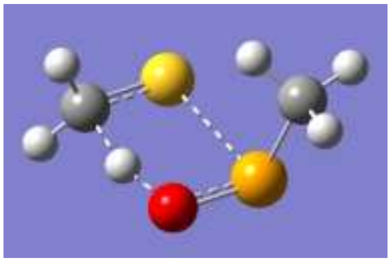

Methyl methanethioselenonate (n = 2; X = S, Y = CH_2)

```
E(RB3LYP) =  -3030.05835639 a.u.; imaginary frequencies = 1

Symbolic Z-matrix:
 Charge =  0 Multiplicity = 1
 C                      1.77596   0.15079   2.28598
 H                      1.63555  -0.38336   3.20243
 H                      1.42744   1.15577   2.40191
 H                      2.81599   0.16071   2.03468
 Se                     0.77679  -0.72086   0.86983
 O                     -0.86506  -0.63608   1.01603
 H                     -1.13012   0.11828  -0.2554
 C                     -0.93741   0.92629  -1.70518
 H                     -1.29315   1.93363  -1.6451
 H                     -1.45206   0.43233  -2.5027
 O                      1.35552  -2.21942   0.75667
 S                      0.68595   0.87162  -1.91045
```

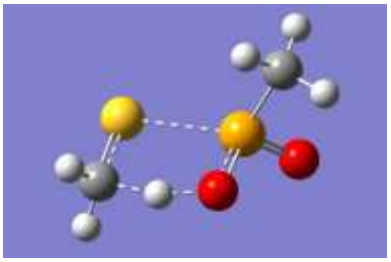

Computations of Zwitterions

Zwitterion 17

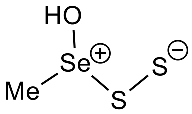


```
E(RB3LYP) =  -3313.79993488 a.u.; imaginary frequencies = 0

Symbolic Z-matrix:
 Charge =  0 Multiplicity = 1
 C                      1.61691  -0.01796   1.37587
 H                      1.9956   -1.00672   1.53033
 H                      1.70986   0.54501   2.28104
 H                      2.17583   0.46075   0.59913
 Se                    -0.25161  -0.11392   0.86301
 O                     -0.89929   1.57713   0.59885
 H                     -0.39782   2.00662  -0.09804
 S                      0.61418  -0.35717  -2.44755
 S                     -0.44099  -1.2609   -0.98116
```

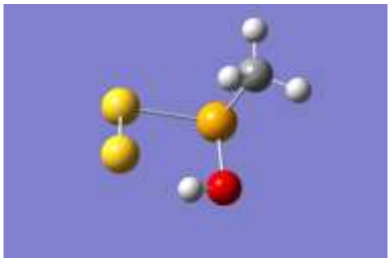

```
Interatomic distances:
Se-S   2.357
S-S    2.047
H-O    1.028
H-S    2.028
Se=O   1.741
```

Zwitterion 18

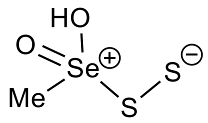

E(RB3LYP) = -3388.98356531 a.u.; imaginary frequencies = 0

Symbolic Z-matrix:
Charge = 0 Multiplicity = 1

C	0.61989	0.81559	1.2419
H	0.97646	0.20674	2.0463
H	-0.15096	1.46512	1.6008
H	1.42676	1.4007	0.85267
Se	-0.09193	-0.31899	-0.16153
O	-1.43098	-1.0119	0.77717
S	-1.16872	1.35736	-1.40766
S	-2.9961	0.82229	-0.57053
O	0.7172	-1.75462	-0.43207
H	-2.25544	-0.24302	0.36594

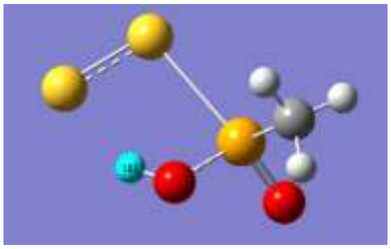

Interatomic distances:

Se-S	2.835
S-S	1.960
H-O	0.976
H-S	2.537
Se=O	1.790,1.618

Table S1. Thermochemistry

All computations were carried out for the gaseous state at T = 298 K.

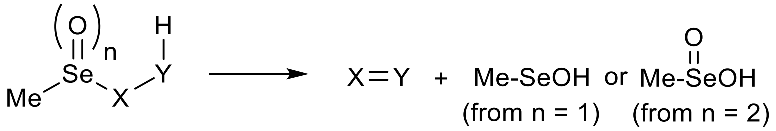

Entry	Reaction N, X, Y	ΔH‡ kcal mol^{-1}	ΔS‡ cal mol^{-1} T^{-1}	ΔG‡ kcal mol^{-1}	ΔH kcal mol^{-1}	ΔS cal mol^{-1} T^{-1}	ΔG kcal mol^{-1}
1	1, C, C	17.75	-2.04	18.36	2.55	38.34	-8.88
2	2, C, C	26.70	-2.21	27.36	-10.88	39.61	-22.68
3	1, O, O	21.72	-5.58	23.39	-1.84	36.49	-12.71
4	2, O, O	25.74	0.00	25.75	-20.51	38.95	-32.12
5	1, O, CH_2	22.85	-1.40	23.27	-4.23	37.69	-15.47
6	2, O, CH_2	38.13	-3.75	39.25	-19.54	38.16	-30.91
7	1, NH, NH	7.08	0.00	7.09	2.30	39.60	-9.50
8	2, NH, NH	12.23	0.54	12.07	-14.04	40.85	-26.21
9	1, CH_2, NH	5.13	-1.15	5.47	1.16	40.47	-10.90
10	2, CH_2, NH	7.64	-6.96	9.71	-13.37	35.44	-23.93
11	1, NH, CH_2	20.14	-1.50	20.59	-1.78	39.53	-13.56
12	2, NH, CH_2	32.95	-3.01	33.85	-16.14	40.01	-28.06
13[a]	1, S, S	2.12	-5.08	3.63	2.05	-2.98	2.94
14	2, S, S	10.82	-2.25	11.49	-14.18	36.30	-24.99
15	1, S, CH_2	14.68	-3.05	15.59	14.67	37.17	3.60
16	2, S, CH_2	23.07	-4.13	24.30	-4.75	37.97	-16.06

a) The data in entry 13 is based on formation of the zwitterion **17.**

Table S2. Electron Density in Transition States

The sums of Mulliken electron charges are indicated for the XY and seleninic or selenonic acid components of transition states.

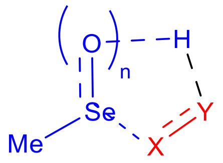

Entry	n, X, Y	$MeSeO_nH$	X=Y
1	1, CH_2, CH_2	-0.003983	0.003985
2	2, CH_2, CH_2	0.094952	-0.094952
3	1, O, O	0.533308	-0.533308
4	2, O, O	0.285414	-0.285414
5	1, O, CH_2	0.250250	-0.250252
6	2, O, CH_2	0.219974	-0.219973
7	1, NH, NH	0.221386	-0.221386
8	2, NH, NH	0.233558	-0.233559
9	1, CH_2, NH	0.214359	-0.214360
10	2, CH_2, NH	0.194970	-0.194971
11	1, NH, CH_2	0.242213	-0.242213
12	2, NH, CH_2	0.217378	-0.217378
13	1, S, S	0.223839	-0.223840
14	2, S, S	0.127481	-0.127482
15	1, S ,CH_2	0.173316	-0.173317
16	2, S, CH_2	0.202401	-0.202402
